# Correlations and Variations Between the Major Biochemical Parameters of the Blood of Hybrid Swine

**DOI:** 10.3390/ani14203002

**Published:** 2024-10-17

**Authors:** Sergei Yu. Zaitsev, Oksana A. Voronina, Nikita S. Kolesnik, Anastasia A. Savina, Aloyna A. Zelenchenkova

**Affiliations:** Federal Research Center for Animal Husbandry Named after Academy Member L.K. Ernst, Dubrovitsy 60, 142132 Podolsk, Moscow Region, Russia; voroninaok-senia@inbox.ru (O.A.V.); kominisiko@mail.ru (N.S.K.); kirablackfire@mail.ru (A.A.S.); aly4383@mail.ru (A.A.Z.)

**Keywords:** genetic-phenotypic correlations, hybrid equation, dispersion model, three-breed swine hybrids, amino acids, blood biochemistry

## Abstract

In modern animal husbandry, increasing attention is given to mathematical modeling and statistical methods, especially for evaluating commercial hybrids. Our aim was to evaluate the phenotypic and genetic variability of biochemical parameters of blood serum of the 56 hybrid boars (Large White × Landrace × Duroc) raised in feeding stations (Russia) through mathematical modeling. The particular variances and covariances of traits were calculated using the limited maximum likelihood model and the REMLF90 programs. A narrow range of variability was found for major biochemical parameters in relationship with the “FFG-factor” (“fattening period × final live weight × gain”), including the majority of the metabolites (*p* ≤ 0.05). The highest values of the genetic correlations were observed for the “total protein” parameter with albumins (0.78), globulins (0.94), creatinine (0.99), and enzymes: AST (0.98), ALT (0.80), etc. Phenotypic and genetic relationships showed fairly high correlation coefficients (0.5–0.8). It is important to emphasize that most of the studied amino acids (alanine, arginine, aspartic acid and asparagine, glutamic acid and glutamine, glycine, isoleucine, leucine, serine, threonine, tyrosine, valine) were significantly associated with the “FFG-factor” (*p* ≤ 0.05). The proposed approach provides reliable data on metabolite variability and correlations.

## Simple Summary

Swine breeding is one of the key aspects of global livestock production, with positive growth in pigmeat production from 2017 to 2022 worldwide and in Russia. This is further demonstrated by an increasing total swine amount (from 2017 to 2022) in the world from 776.6 to 784.2 million heads (FAOSTAT, 2023) and in Russia from 23.1 to 27.6 million heads (ROSSTAT, 2023). Commercial breeds like Landrace, Duroc, and Large White are popular in the USA and Europe, while in Russia, three-breed hybrids (Large White × Landrace × Duroc) are commonly used for commercial farming, according to swine farm management. Based on well-known statistical approaches, we propose for the first time to use a set of such features as the “FFG-factor” (“fattening period × final live weight × gain”), in the equation model as a vector of fixed “phenotypic” effects. In addition, we will try to trace the relationship between the “FFG factor” and biochemical parameters (including amino acids), which is potentially useful for assessing the health status of swine.

## 1. Introduction

Mathematical modeling of biological systems (BioSys) is an important field in both fundamental and applied aspects [1,2,3]. Today, in modern animal husbandry increasing attention is paid to mathematical modeling and statistical methods [4,5,6], especially in the fields of genetic technologies and economic processes in agriculture [7,8,9,10]. Such modeling is known to be based on “cause-and-effect relationships of elements” within the modeled system [1,2,3]. One of the most important findings in mathematical modeling theory and practice (particularly regarding genetic relationships among farm animals) was made by Henderson and coworkers [11]. The methods of genomic forecasting in animal husbandry actively utilize the Henderson’s model [11,12], represented by the Equation (1):

y = Xβ + Zu + є (1)

This model consists of a vector of phenotypes, where “X” is the incidence matrix linking fixed effects to individuals, and “Z” is the genotype matrix. The parameters estimated from the model [12] and Equation (1) include: “β”—the vector of fixed (“nongenetic” or “phenotype” [13]) effects; “u”—the vector of marker (genetic) effects; and “є”—the vector of residuals or errors. The marker effects (vector “u” [11,12] or sometimes “g” [13]) indicate the random partial regression coefficients for each of these effects [11,12,13,14].

The original ideas of the methods and equations, proposed by Henderson and coworkers [11], focus on the possibility of predicting the breeding value (EBV) of the farm animal fathers based on the productivity of offspring [12]. A detailed description of Henderson’s model (i.e., details of the mathematical modeling theory and practice), including a calculation using the best linear unbiased predictor (BLUP), the best linear unbiased estimators (BLUE), and related methods [11,12], are summarized in the “Supplementary materials” section (Table S1). Henderson’s method offers pronounced advantages over the selection index method, especially for swine and sheep breeders [11,12,13,14]. For example, BLUP equations based on Henderson’s method [12] enable the solution of the obtained matrixes and their inverting forms by standard computational calculations.

Examples include describing phenotypes through DNA markers and SNP (multiple pregnancy, stress resistance) [13]; solving the problem of optimizing the feed ration taking into account the following limitations: balance of nutrients; dry matter content, content of trace elements and vitamins, and the specific gravity of feed groups in the ration, etc., [14]. The most commonly used programs in this type of work are the BLUPF90 family [15,16] using REMLF90; limited maximum likelihood methodologies—REML/BLUP forecasting combined with sequential path analysis, etc., [15,16,17]. The construction of mathematical models for biological systems is made possible through the exceptionally intensive analytical work of various experimenters: morphologists, biochemists, physiologists, molecular biologists, etc., [13,18,19,20,21].

As a result of these works, some morphological-functional metabolic schemes and models were proposed, within which various genetic and biochemical processes are represented in an orderly manner in space and time, forming very “complex interweaving” [18,19,20,21]. The main method of design of the complex BioSys models is a computer experiment [14,20,21,22,23]. These requirements imply the development of general and special theories, methods, algorithms, and technologies of computer modeling within the framework of various areas of mathematical biology and husbandry [24,25,26,27,28].

In modern swine farming special attention is given to interbreeding and hybridization due to the following reasons: (a) crossbreeds exhibit higher productivity and vitality compared to purebred animals; (b) two- and three-breed young animals are superior to purebred ones in susceptibility to various diseases; (c) crossbreeding increases the resistance of swine to environmental factors; (d) crossbreeds shows a more efficient fattening that also helps to increase the productivity of sows [13,14,15,16].

The use of interbreeding makes it possible to improve the productive qualities of swine and helps to increase the level of protein metabolism [23,24,25]. For instance, two- and three-breed young swine show increased total protein and albumin content in blood serum compared to purebred animals [16]. Biochemical studies of animal blood play a crucial role in objectively assessing metabolic processes [28,29,30], economically useful traits [14,17], and health status in animals [28]. The study of blood parameters is a way to make an objective intravital assessment of the intensity and direction of metabolism, the course of physiological processes in the body, the level of nutritional value of animals, and their health status [28]. Blood is a liquid matter that creates the internal fluid of the body, necessary for the optimal functioning of the cytological and histological systems of the body. The blood components are biologically active substances that provide blood viscosity, create colloid-osmotic pressure, maintain acid-base balance, provide transport and protective functions, etc. [28].

Physiological homeostasis in farm animals is maintained through a complex system of adaptation mechanisms that counteract harmful factors from the external and internal environments. The composition and physiological-biochemical properties of the internal environment in animals are regulated despite changes in the external environment and shifts in organ and tissue activity [20,28]. Some publications [20,21,22,27,28,29,30] reported that the biochemical parameters of various proteins, carbohydrates, lipids, macro- and microelements, amino acids in the blood of various swine depended on the diet, fattening days, the multiple environmental effects, etc.

A study by Lingaas F. et al. [31] on heritabilities for 20 biochemical and immunological blood parameters was performed based on blood samples from swine at a performance testing station belonging to “the Norwegian Swine Breeders’ Association” [31]. The medium-sized or high estimates of heritability for some of the blood parameters related to disease resistance are encouraging as far as indirect selection is concerned because of “practical difficulties in the recording of some diseases in swine herds” [31]. The other study by Lingaas F. et al. [32] showed pronounced variations in repeatability for different blood parameters although several parameters had moderate or high repeatability, i.e., “the repeatability varied from 0.06 (creatine kinase) to 0.66 (selenium)” [32]. Several blood parameters were influenced by breed (“alanine aminotransferase, alkaline phosphatase, amylase, calcium, C3, gamma glutamyl transferase, globulin, IgG, lactate dehydrogenase, lysozyme, magnesium, selenium and total protein”). Thirteen of the blood parameters were influenced by litter number, and fifteen were influenced by the time of blood sampling after farrowing [31]. The authors concluded that many blood parameters “may possibly have moderate or high heritability” [31,32].

In the study by Elbers et al. [33], the blood samples were taken from 20 swine per herd at the end of the finishing period to investigate hematological and clinical-chemical profiles. The authors found a significant difference in serum albumin concentration “between the low and high lesion prevalence groups” [33]. Differences between castrated males and gilts were demonstrated with respect to mean values of the “blood variables hemoglobin, iron, copper, β-globulin, eosinophil sand segmented neutrophils” [33]. It is important to highlight that the “swine with a higher albumin concentration, a lower gammaglobulin concentration, a higher copper concentration and a higher creatine kinase activity in serum showed a higher daily weight gain” [33].

The authors in [34] proved the hypothesis that the use of some natural ingredients in swine diet formulation associated with improved rearing conditions, enhances the growth performance, digestibility, and selected biochemical and hematological parameters of “Bandjock Local swine” and hybrid “Duroc × Large White swine” [34].

The authors [35] investigated seasonal variation (summer and winter) in some hematological-biochemical parameters of “Zovawk swine at three different age groups”: pre-weaning, grower and adult swine (“reared in the livestock farm of College of Veterinary Sciences and Animal Husbandry Selesih, Aizawl, Mizoram, India”) [35]. The influence of season on the hematological-biochemical profile was most evident “during the grower stage, followed by adults and pre-weaning swinelets” [35].

Additional data can be obtained from the biochemical analysis of swine meat (especially, *Musculus longissimus*) that is presented in the works of [27,28,29,30] and other research groups [20,21,22,31,32,33,34,35,36,37,38]. For example, in the recent paper of Mikolaychik I. et al. [36], the protein quality parameter was significantly higher in the muscle tissue of young swine of the third experimental group (with high iodine concentration) “amounted to 10.90 (*p* < 0.05), which is 1.12% higher than in the muscle tissue of the control group” [36]. The maximum content of oleic acid was found in the lard of animals of the third experimental group—“49.59, which is 1.28% (*p* < 0.05) higher than in the control group” [36].

In the work of Brunes L.C. et al. [37], a weighted single-stage genome-wide association study was conducted to identify genomic regions and putative candidate genes associated with various traits in cattle: “residual feed intake, dry matter intake, feed efficiency, feed conversion ratio, residual body weight gain, residual intake, and weight gain” [37]. The authors identified several protein-coding genes that “explained more than 0.5% of the additive genetic variation in these traits” [37]. These genes were associated with “insulin, leptin, glucose, protein, and lipid metabolism; energy balance; heat and oxidative stress; bile secretion; satiety; feeding behavior; salivation; digestion; and nutrient absorption” [37]. However, no objective biochemical parameters were assessed here [37], unlike in numerous other publications [31,32,33,34,35,36,38].

Based on well-known statistical approaches [11,12,13], we propose for the first time to use a set of such features such as the “fattening period, final live weight and weight gain” (“FFG-factor”) in the equation model as a vector of fixed “phenotypic” effects. In addition, we will try to trace the relationship between the “FFG-factor” and biochemical parameters, including amino acids, which could be useful for assessing production data and swine health.

Therefore, understanding the nature and patterns of variability of biochemical indicators in swine blood is of particular interest.

The aim of this work is to evaluate the phenotypic and genetic variability of biochemical parameters in the blood serum of the three-breed hybrids boars through mathematical modeling with the particular “FFG”-included equation.

## 2. Materials and Methods

### 2.1. Samples of the Swine Blood Serum

The studies were carried out on boars (n = 56 total animals) of the three-breed hybrids (Large White × Landrace × Duroc) grown at the feeding stations of the selection-hybrid center (Russia). Data on the basis of which the decision was made to include in the group: animal identification number, breed, sex, date of birth, date of putting on fattening, date of removing from fattening, data on weight gain, live weight. The living weight and age ranges of the experimental animals were 105 ± 5 kg (at the moment of slaughter) and approximately 5 months. Before the main calculations, the normality of distribution by weight (at the beginning and at the end of fattening), fattening period and age were checked. The main diet (Supplementary Materials, Tables S2–S4) and conditions of keeping animals (temperature, humidity, light conditions and gas composition of the air in the room) during the studied periods were the same and within the limits of the national zoohygienic standards [28,29,30]. Blood was collected from the tail vein at the end of fattening. Serum was separated using centrifugation for 15 min at 3000 rpm (laboratory centrifuge SM-12, Russia). The experiments were carried out in compliance with the requirements set out in the Directive of the European Parliament and of the Council of the European Union 2010/63/EU of 22 September 2010 on the protection of animals used for scientific purposes and the principles of treatment of animals, in accordance with Article 4 of the Federal Law of the Russian Federation N 498-FZ [28].

The experimental protocols (concerning these animals) were approved by the Ethics Committee of the Federal Research Center for Animal Husbandry named after Academy Member L.K. Ernst (protocol code: 2023–2303; date of approval: 23 March 2023). All experiments and conditions (animal care, feeding, biological material sampling, etc.) were fulfilled in accordance with the applicable regulations (internationally recognized guidelines and local acts).

The main biochemical and amino acid parameters of the blood serum of all these hybrids were obtained and analyzed (Section 2.2 and Section 2.3).

### 2.2. Measurements of the Biochemical Indicators of Swine Blood Serum Samples

The biochemical indicators of animal blood serum sample were determined using a “ChemWell” automatic biochemical analyzer (Awareness Technology, Palm City, FL, USA) with reagents of “Analyticon Biotechnologies AG” (Lichtenfels, Germany) and “Spinreact” (Barcelona, Spain). Biochemical indicators [28,29,30,39] such as the concentration of total protein (TP)—using the biuret method; albumin (A)—using the colorimetric method; urea—using the enzymatic colorimetric (Berthelot method); creatinine—using the kinetic Yaffe method; glucose—using the enzymatic glucose oxidase method; cholesterol (Chol) and triglycerides (TG) using the enzyme-colorimetric method; bilirubin (quantification using the Walters and Gerarde method); calcium (Ca) using the O-cresolphthalein complexon method; phosphorus (P), magnesium (Mg) and iron (Fe) using the colorimetric method; alanine aminotransferase (ALT) activity using the UV-kinetic method; aspartate aminotransferase (AST) activity using the UV-kinetic method; alkaline phosphatase (ALP) activity using the kinetic method were determined [28,29,30]. The ratios and indicators A/G, Ca/P, ALT/AST and the concentration of globulins (G) were determined through calculation [28,29,30]. The results of these measurements were statistically processed using the “R” program.

### 2.3. Measurements of the Amino Acid Content of Swine Blood Serum Samples

Exactly 200 μL of each sample of the swine blood serum was selected and added to 3 mL of 6 N hydrochloric acid (HCl) for complete hydrolysis according to the paper [29]. The hydrolysis was carried out in fluoroplastic beakers with a screw cap (CEM, Corporation, Matthews, NC, USA), in a thermostat at 110 °C for 24 h. Norleucine was used as an internal standard for AA analysis. During this hydrolysis (6 H HCl, 24 h, 110 °C), tryptophan was completely destroyed, and to determine cysteine and methionine, the samples were pre-oxidized by adding 500 µL performic acid. After hydrolysis, 160 μL of the resulting suspension was taken and evaporated at 110 °C to remove hydrochloric acid. Then, 1 mL of sample dilution buffer was added. The resulting suspension was centrifuged at 13,000 rpm for 5 min. The final amino acid analysis was carried out using an LC-20 Prominence high-performance liquid chromatography system (Shimadzu, Tokyo, Japan) equipped with a reaction module for post-column derivatization with ninhydrin ARM-1000 (Sevko & Co., Moscow, Russia) and a column with ion exchange resin 4.6 × 150 mm (Sevko & Co., Moscow, Russia). It is worth noting that under these analytical conditions, the amino acids asparagine (ASN) and glutamine (GLN) are not separated on the chromatogram from ASP and GLU. That is why they are defined as ASP + ASN and GLU + GLN, respectively [29].

### 2.4. Mathematical Modeling and Calculations

The correlations between all parameters of the swine blood serum were fulfilled using statistical treatment by “R” program. Based on the database of the studied parameters, the authors applied the hybrid equation model (2) using the REMLF90 procedure to obtain variance and correlation values during the calculation of the parameters [40,41]. To calculate the values of particular variances and covariances of traits, the method of limited maximum likelihood was used according to the model using the REMLF90 programs. This is represented by the following Equation (2):

(2) $$Y_{ilk\;}=\mu +FFG_{i\;}+Sire_{l}+e_{ilk},$$

where *Y_ilk_*—the estimated indicator of the *k*th boar; *μ*—the population constant; *FFG_i_*—the fixed effect of the *i*th “fattening period × final live weight × gain”; *Sire_l_*—the randomized effect of the *l*th producer (*l* = 1, … heads); *e_ilk_*—the effect of unaccounted factors. To calculate the fixed “FFG-factor”, we first performed a ranking procedure from smallest to largest in the following sequence: fattening period (in days), final live weight (in kg with a step for the rank of 1 kg), gain (g with a step for the rank of 50 g). Thus, the fixed “FFG-factor” is a categorical characteristic of the animals under consideration depending on the fattening period in days, final live weight and gain.

To determine the strength of the influence of the fixed effect “fattening period × final live weight × gain” (coded into 1st factor according to the HYS principle), we used ANOVA. Let *x_ik_* denote the value of the *k*th quantity in the *i*th group. The Equation (3) of the one-factor ANOVA model can be represented as:

(3) $$x_{ik}=a+m_{i}+\varepsilon_{ik},\;$$

where *a*—the general average of all observation results, i.e., *M(X)*, *m_i_*—the effect of influence on *X* caused by the *i*th level of factor *A*, in other words, the deviation of the particular mathematical expectation *a_i_* of the resulting feature at the *i*th level of the factor from the general mathematical expectation *a*, i.e., m_i_ = *a_i_*− *a*; *ɛ_ik_*—a random residue reflecting the influence of all other uncontrolled factors on the value *x_ik_*.

## 3. Results and Discussion

### 3.1. Biochemical Indicators of the Blood Serum of Swine Hybrids

Based on the statistical analysis of the measured indicators of the amino acid and biochemical composition in the blood serum of swine hybrids, the following data (Table 1) were obtained.

All analyzed blood parameters of the studied hybrids had values within “physiologically acceptable intervals” (so-called PAIs) [29,30,31,32,33,34,35,36,37,38,39]. At the same time, the total bilirubin value was close to the lower limit of PAIs, and the alkaline phosphatase value was close to the upper limit of PAIs.

Biochemical parameters of the blood of the studied hybrids, such as total protein (77.99 g/L), albumins (39.23 g/L), globulins (37.04 g/L), urea (5.48 mM/L), Bil-T (1.18 μM/L) (Table 1) had average values that exceed similar values for purebred swine (Landrace, Duroc, Large White or Chester White, etc.) and their two-breed hybrids obtained by Russian [28] and international groups of authors [31,32,33,34,35]. For example, the average values for the total protein (albumin) in the blood obtained for 4–7 month-old swine of Russian selection from different farms [28]: 67.21 (37.48) g/L (Large White), 76.4 (38.9) g/L (Duroc), 69.32 (37.99) g/L (Large White × Duroc), 70.01 (39.00) g/L (Large White × Yorkshire), or for swine of similar age of foreign selections: 65.98 (37.04) g/L (Norwegian Landrace or Norwegian Yorkshire) [31,32], 67.4–67.8 (34.5–34.7) g/L (“unknown purebred swine, housed and managed under normal field conditions in The Netherlands”) [33], etc. However, the “reference values for blood variables from swine” given by Kaneko J.J. [38] are 79.0–89.0 g/L (“unknown purebred swine”), i.e., generally somewhat higher than those in Table 1. Thus, the average values we obtained for protein indices (total protein, albumins, globulins, and others) in the blood of three-breed hybrid swine aged 5–7 months of Russian selection are in the PAIs range and indicate a more active protein (amino acid) metabolism, compared to purebred swine and their two-breed hybrids, which is certainly positive, especially in the period before slaughter.

On the other hand, there is insufficient information in the literature on average values for lipid indices for swine in the period before slaughter. For example, the average values for TG and Chol in the blood of swine ~5 months of foreign selection are 0.45 and 2.53 mM/L (Norwegian Landrace or Norwegian Yorkshire) [31,32], etc. However, among the “reference values for blood variables from swine” given by Kaneko J.J. [38], there are values only for Chol 0.93–1.40 g/L (“unknown purebred swine”), i.e., in general, somewhat higher than those in Table 1. Thus, the average values obtained by us for lipid parameters in the blood of three-breed hybrid swine 5 months of Russian selection indicate a less active lipid metabolism, compared to purebred swine, which can lead to a slight decrease in fat deposition in the period before slaughter and is in demand by consumers who prefer less fatty pork.

Most of the other parameters change insignificantly (within the PAI range) and will not be discussed here. There is a vast amount of data on the biochemical parameters of the blood of purebred swine and their hybrids, aged from 1 day to 1–2 months, which we will not discuss since these parameters differ significantly from those for swine aged 5–6 months, which are more relevant for pork consumers.

### 3.2. The Parameters of Variability and Stability of the Distribution of the Biochemical Indicators of the Blood Serum of Swine Hybrids

As for the parameters determining the variability and stability of the distribution of these sample values, no significant deviations were found here. The median, representing the middle value of the set of numbers, almost coincides with the average statistical value. Regarding the standard deviation (σ) and the coefficient of variation (C_v_), among the biochemical parameters, the following were characterized by the greatest share of variability: globulin, glucose, bilirubin, albumin-globulin coefficient, alanine aminotransferase (ALT), aspartate aminotransferase (AST), magnesium, etc. In general, amino acid values are more balanced in their variability values compared to the biochemical parameters.

All analyzed amino acid parameters of the studied blood samples had values within the PAIs for the specified swine hybrids (Table 1). As for the indicators determining the variability and stability of the distribution of data values of the sample, including standard deviations (σ), no significant deviations were found here. As for the coefficient of variation or relative standard deviation, among the amino acid indicators, the following were characterized by the greatest share of variability: MET > SER > PRO > CYS > THR > GLY > VAL > ARG > TYR > HIS > ILE > GLU + GLN > ALA > PHE > LYS > LEU > ASP+ ASN. Regarding the maximum distribution of random variables of the indicators and the excess coefficient (kurtosis), almost all data had minor deviations, with the exception of the CYS and MET indicators, which is due to the instability of their ratio and relatively low content in the blood of the studied swine hybrids.

For the hybrid model equation, we selected “fattening period × final live weight × gain” (FFG) as a fixed factor and determined the degree and direction of its influence on the studied indicators using variance analysis. Usually, in mathematical modeling and studies on some farm animals, when using linear equations and the BLUP approach, “HYS” (“Herd—Years—Season” [11,12]) is used as a fixed factor that is especially suitable for cattle [40,41,42]. We chose swine hybrids as the object of our research. That is why, we considered that “FFG-factor” can be more reasonable as a fixed effect. As a result, the influence of the complex “FFG-factor” (not only each of the three factors separately), as well as all possible interactions between them can be taken into account. We used dispersion analysis to determine the correctness of our approach and all our calculations based on the hybrid model equation. In our opinion, the dispersion analysis can be the best for complete and correct characterization of the degree of relationship between the “FFG-factor” and blood biochemical parameters. This allows us to evaluate also the determination coefficient and the strength of the “FFG-factor’s” influence as an element of our model (Table 2).

There is a positive reliable relationship between the “FFG-factor” and a number of biochemical indices, as well as the amino acid composition of blood proteins in accordance with the variance analysis procedure (including the Fisher criterion). The high degree of determination according to the coefficient (R^2^) indicates a high influence of the “FFG-factor” on such indices as total protein, albumin, globulin, A/G, urea, creatinine, glucose, bilirubin, cholesterol, ALT and AST, Ca, P, Ca/P and chlorides. As one can see, a significant number of biochemical indicators are subject to the reliable influence of this factor (Figures S1 and S2 in the Supplementary Materials). Among amino acids, a large number of them are also subject to the influence of the “FFG-factor”: aspartic acid (+asparagin), threonine, serine, glutamic acid (+glutamin), glycine, alanine, valine, isoleucine, leucine, tyrosine, arginine, proline (Figure S3 in the Supplementary Materials).

There is a positive reliable relationship between the “FFG-factor” and a number of amino acid indices, as well as the amino acid composition of blood proteins, according to the Fisher criterion (F) and in accordance with the variance analysis procedure. The high degree of determination according to the coefficient (R^2^) indicates a high degree of influence of the “FFG” factor on such amino acid indices as SER > ARG > GLY > TYR > GLU + GLN > VAL > ASP+ ASN > ALA > LEU > ILE > THR. On the other hand, the degree of influence of these factors on such amino acid indices as CYS, MET, PHE, PRO, LYS, HIS is unreliable (Table 2).

When considering 3 cationic AAs (ARG (*p* < 0.05), LYS (*p* > 0.05), HIS (*p* > 0.05) in the context of the relationship with the “FFG factor”, important comments can be made. It is well-known that ARG is formally an essential AA, but it participates in the “urea cycle”. Therefore, for an adult organism, ARG can be taken from the “urea cycle” and used for protein synthesis in case of its deficiency in the diet. The degree of its “indispensability” is closely related to both the diet and age, since the level of ARG synthesis in the body increases with age. Thus, ARG is closely related to the “FFG-factor”, explaining the high reliability. Lysine, in turn, is not only an essential AA, but also a limiting AA for swine, and therefore this indicator changes even with a slight imbalance in the AA composition of the feed. This may be the reason for the low reliability of the relationship between LYS and the “FFG-factor”.

Histidine is a conditionally replaceable AA for most mammals. Its amount is determined mainly by the activity of histidinol dehydrogenase, which oxidizes histidinol to histidine. The activity of histidinol dehydrogenase is an indicator by which the indispensability of histidine for a particular animal species can be judged [42,43]. When comparing the activity of histidinol dehydrogenase from the liver and kidneys of cattle with similar activity in swine, it was found that the amount of histidine synthesized cannot satisfy the swine’s need for it [44]. Another feature of His metabolism in swine is that some of it, in non-protein form (methylated HIS), is stored in skeletal muscles in the form of the dipeptide balenine, is not re-incorporated into proteins, and is excreted in the urine. Therefore, the histidine content in body proteins and the need for histidine, calculated from its content in the body, may not coincide in swine [28].

Despite proline being a replaceable amino acid and being contained in all body proteins, it has a high degree of variability and, as a result, the lowest reliability of the relationship (*p* = 0.159). This is likely due to the peculiarity of the structure of this amino acid. In proteins, the nitrogen atom in the proline molecule is not bonded to the hydrogen atom, so, the X-PRO peptide group cannot be a hydrogen donor when forming a hydrogen bond. With its conformation rigid structure, proline strongly bends the peptide chain. PRO is a “rotary” amino acid and is actively involved in the formation of the secondary structure of the protein. Therefore, its content strongly depends on the amount, ratio, and conformation of proteins, leading to the low reliability of the relationship and the high variability of this indicator. Sulfur-containing amino acids, due to the formation of “disulfide bridges”, also play a large role in the assembly of proteins (tertiary structure), so their amount also strongly depends on the ratio of protein fractions, reducing the reliability of the relationship with the “FFG-factor”.

### 3.3. The Parameters of the Genetic and Phenotypic Correlations of the Indicators of the Blood Serum of Swine Hybrids

For a better understanding of the influence of the studied factors on the amino acid (Table 3) and major biochemistry (Table 4) parameters of the blood of swine hybrids, we presented these data as genetic and phenotypic correlations using the hybrid equation of our model (in the REMLF90 BLUPF90 subroutine).

Through computer calculations using a hybrid equation of our model (when applying the REMLF90 BLUPF90 subroutine), we obtained genetic and phenotypic variances, and correlations reflecting the variability of the studied biochemical parameters were obtained (Table 4).

The highest values of the genetic correlation coefficients are observed for biochemical parameters such as the “total protein” parameter, which is reliably positively associated with albumin (0.78), globulin (0.94), creatinine (0.99), glucose (0.59), bilirubin (0.70), triglycerides (0.77), cholesterol (0.78) and AST (0.98). The other correlations were at the level of r = 0.59–0.90. Total protein had a negatively strong reliable relationship with ALT (−0.80), alkaline phosphatase (−0.76), calcium (−0.82), Ca/P (−0.95), iron (−0.93), and chlorides (−0.97). Albumin and globulin show opposite correlation with creatinine (−0.71 and +0.90), glucose (−0.95 and +0.82), bilirubin (−0.90 and +0.86), triglycerides (−0.87 and +0.87), ALT (0.93 and −0.91), and AST (−0.64 and +0.85) [38,39,40]. In this case, albumin is predominantly negative, r = −0.60–0.90, while globulin is positive, r = 0.80–0.90. Glucose, bilirubin and triglycerides, along with several trace elements, have close relationships with each other (Table 4). Using the method of calculating genetic relationships, we obtained a classical interaction of biochemical components in a healthy organism system.

Thus, using the method of calculating genetic relationships, we obtained the classical interaction of biochemical components in the system of a healthy organism.

This is as far as the nature of the genetic relationship is concerned, but as far as the phenotypic relationship is concerned, the picture is somewhat different. Regarding phenotypic relationships, a high phenotypic relationship was established between the total protein/globulin ratio (r = 0.90) and the albumin/globulin ratio (r = −0.80). Amino acids show strong phenotypic and genetic correlations, with aspartic acid and arginine having a strong negative relationship with almost all other amino acids, ranging from r = −0.50–0.90 (Table 3).

The majority of the “proteinogenic” amino acids (13 out of 19) have high values of genetic correlation coefficients (r = 0.50–0.99). In contrast, such amino acids like CYS, MET, ARG and HIS (as well as LYS and PRO, to some extent) have low coefficients (Table 3). Weak dependencies are understandable mainly for sulfur-containing and cationic amino acids from the perspective of structural and functional properties, and biochemistry of metabolic processes. These amino acids and PRO as the multifunctional cyclic “imino acid” (Table 3) have low coefficients in phenotypic correlations. Overall, the study results indicate that genetic factors may play a role in regulating the amino acid composition of blood, potentially due to some amino acids acting as precursors for others or participating in protein formation. In this case, changes in one amino acid level can lead to changes in other amino acid levels through common metabolic pathways.

## 4. Conclusions

Thus, the variability of the studied parameters of the biochemical blood composition is associated to varying degrees with the factor “FFG” (“fattening period × final live weight × gain”). Triglycerides, alkaline phosphatase, magnesium, and iron showed a narrow range of variability in their relationship with this factor. Key metabolites of protein, carbohydrate, lipid, and mineral metabolism significantly changed in relationships with the “FFG-factor” (*p* ≤ 0.05).

Phenotypic and genetic relationships are characterized by fairly high correlation coefficients (0.5–0.8). It is important to emphasize that most of the studied amino acids are fairly associated with the “FFG-factor” (*p* ≤ 0.05), except for CYS, MET, PHE, PRO, LYS, HIS. The low reliability of the relationships of these six amino acids with the “FFG-factor” is likely associated with their particular metabolism in the body, chemical structure, and role in protein formation. The proposed equations provide insights into the nature of metabolite variabilities and patterns of genetic and phenotypic correlations.

## Figures and Tables

**Table 1:** Biochemical indicators of the blood serum of swine hybrids.

Indicators	X	±x	σ	C_v_	Min	Max
	**Biochemical indicators**					
TP, g/L	77.99	0.66	3.69	4.73	68.70	83.60
A, g/L	39.23	0.31	1.68	4.29	35.80	41.60
G, g/L	37.04	0.50	2.69	7.26	33.30	41.20
Urea, mM/L	5.48	0.10	0.53	9.57	4.88	6.49
Crea, μM/L	147.56	1.41	7.76	5.05	136.91	160.08
Gluc, mM/L	2.91	0.07	0.39	13.22	2.27	3.45
Bil-T, μM/L	1.18	0.04	0.27	22.85	0.92	2.27
TG, mM/L	0.29	0.01	0.04	13.41	0.23	0.36
Chol, mM/L	2.22	0.01	0.06	2.84	2.12	2.35
ALT, IU/L	60.86	1.24	6.70	11.00	48.70	70.70
AST, IU/L	54.97	0.71	3.86	7.03	49.40	61.50
ALP, IU/L	139.61	4.22	22.31	15.98	113.00	188.00
Ca, mM/L	3.01	0.03	0.15	4.96	2.76	3.17
Mg, mM/L	1.37	0.02	0.10	7,25	1.23	1.54
Fe, μM/L	26.25	0.34	1.81	6.90	23.40	29.84
Cl, mM/L	103.63	0.16	0.85	0.82	101.40	104.80
	**Amino acid indicators (mg/100 mg)**					
ASP + ASN	0.64	0.007	0.05	7.97	0.51	0.75
THR	0.34	0.005	0.04	11.79	0.26	0.42
SER	0.39	0.011	0.08	21.54	0.23	0.61
GLU + GLN	1.04	0.013	0.10	9.41	0.80	1.24
GLY	0.28	0.004	0.03	11.47	0.21	0.34
ALA	0.46	0.005	0.04	8.81	0.36	0.52
CYS	0.21	0.003	0.03	12.47	0.16	0.32
VAL	0.52	0.007	0.05	10.61	0.41	0.62
MET	0.06	0.002	0.01	21.71	0.05	0.09
ILE	0.28	0.003	0.03	9.43	0.22	0.33
LEU	0.83	0.009	0.07	8.22	0.67	0.95
TYR	0.46	0.006	0.05	9.96	0.38	0.56
PHE	0.50	0.005	0.04	8.32	0.42	0.59
HIS	0.31	0.004	0.03	9.60	0.24	0.36
LYS	0.69	0.007	0.06	8.25	0.55	0.80
ARG	0.51	0.007	0.05	10.35	0.39	0.67
PRO	0.41	0.008	0.06	15.07	0.31	0.57

Notes: X is the mean; ±x is the error of the mean; σ is the standard deviation; C_v_ is the coefficient of variation; Min is the minimum value; Max is the maximum value. Biochemical indicators: TP—Total protein, g/L; A—Albumin, g/L; G—Globulin, g/L; A/G—A/G; U—Urea, mM/L; Crea—Creatinine, μM/L; Gluc—Glucose, mM/L; Bil-T—Bilirubin total, μM/L; TG—Triglycerides, mM/L; Chol—Cholesterol, mM/L; alanine transaminase—ALT, IU/L; aspartate transaminase—AST, IU/L; AST/ALT—De Ritis ratio, a.u.; ALP—Alkaline phosphatase, IU/L; Ca—Calcium, mM/L; Phosphorus—P, mM/L; Ca/P—Calcium/Phosphorus, a.u.; Mg—Magnesium, mM/L; Fe—Iron, μM/l; Cl—Chlorides, mM/L. Amino acids (mg/100 mg): ASP is aspartic acid, ASN is asparagine, THR is threonine, SER is serine, GLU is glutamic acid, GLU is glutamine, GLY is glycine, ALA is alanine, CYS is cysteine, VAL is valine, MET is methionine, ILE is isoleucine, LEU is leucine, TYR is tyrosine, PHE is phenylalanine, HIS is histidine, LYS is lysine, ARG is arginine, PRO is proline.

**Table 2:** An influence of the “FFG-factor” on the biochemical and amino acid indicators.

Indicators	Mult. R	Mult. R^2^	Corr. R^2^	F	*p*	
**Biochemical indicators**						
**TP, g/L**	0.71	0.50	0.44	8.449	<0.001	
**A, g/L**	0.59	0.35	0.27	4.478	0.001	
**G, g/L**	0.69	0.48	0.42	7.813	<0.001	
**Urea, mM/L**	0.53	0.28	0.19	3.284	0.008	
**Crea, μM/L**	0.51	0.26	0.18		3.031	0.013
**Gluc, mM/L**	0.47	0.22	0.13		2.378	0.042
**Bil-T, μM/L**	0.49	0.24	0.15		2.718	0.023
**TG, mM/L**	0.36	0.13	0.03		1.291	0.278
**Chol, mM/L**	0.72	0.52	0.46		9.035	<0.001
**ALT, IU/L**	0.65	0.42	0.35		6.108	<0.001
**AST, IU/L**	0.47	0.22	0.13		2.386	0.042
**ALP, IU/L**	0.38	0.15	0.05	1.477	0.205	
**Ca, mM/L**	0.65	0.42	0.36	6.232	<0.001	
**Mg, mM/L**	0.30	0.09	−0.02	0.812	0.565	
**Fe, μM/L**	0.41	0.17	0.07	1.751	0.128	
**Cl, mM/L**	0.58	0.33	0.25	4.236	0.002	
**Amino acid indicators**						
**ASP + ASN**	0.49	0.24	0.15	2.623	0.027	
**THR**	0.46	0.21	0.12	2.293	0.049	
**SER**	0.70	0.48	0.42	7.983	<0.001	
**GLU + GLN**	0.55	0.30	0.22	3.628	0.004	
**GLY**	0.59	0.35	0.27	4.557	<0.001	
**ALA**	0.47	0.22	0.13	2.361	0.043	
**CYS**	0.43	0.19	0.09	1.963	0.088	
**VAL**	0.52	0.27	0.19	3.186	0.009	
**MET**	0.44	0.19	0.10	2.008	0.082	
**ILE**	0.47	0.22	0.13	2.426	0.039	
**LEU**	0.49	0.24	0.15	2.656	0.025	
**TYR**	0.56	0.32	0.24	3.958	0.002	
**PHE**	0.44	0.20	0.10	2.089	0.071	
**HIS**	0.41	0.17	0.07	1.749	0.129	
**LYS**	0.42	0.18	0.08	1.813	0.115	
**ARG**	0.65	0.42	0.35	6.117	<0.001	
**PRO**	0.40	0.16	0.06	1.628	0.159	

Notes: Multiple R is the multiple correlation coefficient, characterizes the closeness of the relationship between the dependent variable and the predictor, “multiple R^2^“ is the coefficient of determination, “adjusted R^2^” is the coefficient of determination adjusted for the number of factors, the F-criterion evaluates the statistical significance of the difference in means in groups (Fisher criterion), *p* is the probability of an erroneous result (*p* below 0.049 or *p* < 0.049). The indicators with a reliable influence of the analyzed factor are highlighted in bold in the table.

**Table 3:** Correlogram of relationships * (genotypic and phenotypic) between the amino acid parameters of the blood of swine hybrids.

	1	2	3	4	5	6	7	8	9	10	11	12	13	14	15	16	17
1	1.00	0.86	0.75	0.84	0.89	0.75	0.60	0.83	0.38	0.76	0.83	0.87	0.80	0.38	0.61	−0.18	0.53
2	−0.73	1.00	0.76	0.83	0.92	0.71	0.56	0.86	0.30	0.71	0.86	0.93	0.86	0.30	0.61	−0.26	0.65
3	0.67	−0.13	1.00	0.79	0.80	0.49	0.56	0.69	0.43	0.47	0.66	0.74	0.56	0.20	0.55	−0.09	0.51
4	0.85	−0.97	0.28	1.00	0.87	0.62	0.69	0.83	0.55	0.77	0.89	0.78	0.71	0.28	0.66	−0.19	0.51
5	0.84	−0.38	0.96	0.53	1.00	0.78	0.66	0.96	0.42	0.81	0.93	0.91	0.86	0.42	0.69	−0.13	0.63
6	0.94	−0.65	0.83	0.76	0.95	1.00	0.51	0.81	0.24	0.77	0.76	0.75	0.81	0.43	0.42	−0.33	0.73
7	0.96	−0.77	0.72	0.86	0.88	0.98	1.00	0.70	0.65	0.63	0.64	0.54	0.49	0.43	0.47	−0.33	0.40
8	0.66	−0.10	1.00	0.25	0.95	0.81	0.70	1.00	0.43	0.87	0.91	0.85	0.84	0.50	0.65	−0.18	0.67
9	0.95	−0.72	0.78	0.82	0.92	0.99	1.00	0.76	1.00	0.43	0.43	0.27	0.25	0.24	0.27	−0.29	0.16
10	0.95	−0.87	0.57	0.94	0.78	0.93	0.98	0.55	0.96	1.00	0.90	0.75	0.79	0.55	0.65	−0.27	0.53
11	0.76	−0.98	0.10	0.98	0.37	0.64	0.75	0.07	0.70	0.87	1.00	0.85	0.87	0.47	0.77	−0.22	0.52
12	0.87	−0.47	0.93	0.60	0.99	0.97	0.92	0.92	0.95	0.83	0.44	1.00	0.92	0.36	0.59	−0.18	0.63
13	0.81	−0.34	0.97	0.49	1.00	0.93	0.86	0.96	0.90	0.75	0.33	0.99	1.00	0.39	0.56	−0.16	0.68
14	0.95	−0.73	0.76	0.83	0.91	0.99	1.00	0.75	1.00	0.96	0.71	0.94	0.89	1.00	0.65	−0.13	0.15
15	0.79	−0.98	0.16	0.99	0.43	0.68	0.80	0.14	0.74	0.90	0.99	0.50	0.39	0.76	1.00	0.00	0.13
16	−0.94	0.69	−0.80	−0.79	−0.93	−0.99	−0.99	−0.78	−1.00	−0.94	−0.66	−0.96	−0.91	−0.99	−0.71	1.00	−0.21
17	0.37	0.24	0.93	−0.09	0.79	0.57	0.42	0.94	0.49	0.24	−0.27	0.74	0.82	0.48	−0.20	−0.53	1.00

Notes: 1—aspartic acid, 2—threonine, 3—serine, 4—glutamic acid, 5—glycine, 6—alanine, 7—cysteine, 8—valine, 9—methionine, 10—isoleucine, 11—leucine, 12—tyrosine, 13—phenylalanine, 14—histidine, 15—lysine, 16—arginine, 17—proline. * Below the diagonal are genetic correlations; above are phenotypic correlations.

**Table 4:** Correlogram of relationships * (genotypic and phenotypic) between the major biochemical parameters of the blood of swine hybrids.

	1	2	3	4	5	6	7	8	9	10	11	12	13	14	15	16
1	1.00	0.31	0.97	−0.19	0.45	0.17	0.50	0.16	0.42	0.36	0.36	0.28	0.18	0.40	0.31	0.34
2	0.78	1.00	0.19	0.27	0.31	0.38	0.41	0.08	0.64	0.36	0.27	0.82	0.40	0.11	0.44	0.81
3	0.94	−0.94	1.00	−0.17	0.47	0.19	0.46	0.12	0.35	0.36	0.41	0.22	0.15	0.39	0.31	0.33
4	0.25	0.40	−0.09	1.00	0.50	0.27	0.08	−0.09	0.40	0.23	0.28	0.21	0.23	0.10	0.17	0.25
5	0.99	−0.71	0.90	0.35	1.00	0.37	0.43	0.05	0.50	0.35	0.43	0.17	0.14	0.40	0.30	0.42
6	0.59	−0.95	0.82	−0.64	0.50	1.00	0.40	0.53	0.45	0.44	0.46	0.41	−0.15	−0.20	0.60	0.55
7	0.70	−0.90	0.86	−0.42	0.63	0.90	1.00	0.55	0.37	0.43	0.51	0.33	−0.24	0.20	0.47	0.33
8	0.77	−0.87	0.87	−0.24	0.71	0.82	0.87	1.00	0.12	0.22	0.34	0.13	−0.70	−0.23	0.40	0.11
9	0.78	−0.22	0.52	0.80	0.84	−0.05	0.15	0.32	1.00	0.59	0.15	0.68	0.30	−0.06	0.38	0.44
10	−0.80	0.93	−0.91	0.27	−0.74	−0.85	−0.81	−0.81	−0.32	1.00	0.28	0.48	0.00	0.09	0.50	0.37
11	0.98	−0.64	0.85	0.44	0.99	0.41	0.55	0.67	0.89	−0.68	1.00	0.10	0.01	0.32	0.39	0.54
12	−0.76	1.00	−0.93	0.43	−0.69	−0.96	−0.91	−0.86	−0.19	0.93	−0.61	1.00	0.23	−0.18	0.50	0.65
13	−0.82	0.99	−0.96	0.34	−0.75	−0.94	−0.91	−0.90	−0.28	0.93	−0.69	0.99	1.00	0.36	−0.06	0.33
14	0.53	0.10	0.22	0.95	0.61	−0.37	−0.15	0.03	0.94	−0.02	0.69	0.14	0.04	1.00	0.05	0.20
15	−0.93	0.95	−0.99	0.11	−0.88	−0.83	−0.85	−0.86	−0.50	0.93	−0.84	0.94	0.97	−0.19	1.00	0.53
16	−0.97	0.89	−0.98	−0.05	−0.94	−0.73	−0.79	−0.83	−0.63	0.89	−0.91	0.88	0.92	−0.35	0.98	1.00

Notes: 1—Total protein, g/L; 2—Albumin, g/L; 3—Globulin, g/L; 4—Urea, mM/L; 5—Creatinine, μM/L; 6—Glucose, mmol/L; 7—Total bilirubin, μM/L; 8—Triglycerides, mmol/L; 9—Cholesterol, mM/L; 10—ALT, IU/L; 11—AST, IU/L; 12—Alkaline phosphatase, IU/L; 13—Ca, mM/L; 14—Mg, mM/L; 15—Fe, μM/L; 16—Chlorides, mM/L. * Below the diagonal are genetic correlations; above are phenotypic correlations The values (shown in Table 3) present the average amino acid composition in swine blood, divided by the levels of influence of the corresponding factors. Most of the measured parameters have a relatively uniform distribution, ranging from ±0.10 to ±0.49, but some of them are reaching higher values (ranging from ±0.50 to ±0.99). This allows for a clear assessment of the diversity of influence on the amino acid profile of blood proteins (Table 3).

## Data Availability

The data supporting reported results can be found at https://www.vij.ru/institut/struktura-organizatsii/nauchnye-podrazdeleniya/gruppa-analiticheskoj-biokhimii-2 (accessed on 1 August 2024).

## Supplementary Materials

The following supporting information can be downloaded at https://www.mdpi.com/article/10.3390/ani14203002/s1, and included the following: Table S1. Description of the models. Table S2. Structure of the SK-5 diet, which was received by the swinelets, who reached a live weight of 20–25 kg (they received the SK-5 diet when put on rearing). Table S3. Structure of the SK-6 diet, which was received by boards with a live weight from 28 to 30 kg to 70 kg, (the first period of their fattening). Table S4. Structure of the SK-7 diet, which was received by boards with a live weight from 70 to 105 kg (the second period of their fattening). Figure S1: Decomposition of average values of some biochemical parameters depending on the influencing factor (FFG). Figure S2: Decomposition of average values of some biochemical indicators depending on the influencing factor (FFG). Figure S3: Decomposition of the average values of the amino acid composition of blood proteins depending on the influencing factor (FFG).
