# Supplementary material for: Correlations and Variations Between the Major Biochemical Parameters of the Blood of Hybrid Swine
Source: Animals (Basel). 2024 Oct 17;14(20):3002. doi: 10.3390/ani14203002 (PMC11504049; doi:10.3390/ani14203002)
Supplement: Supplementary file 1 [file animals-14-03002-s001.zip › animals-3163191-supplementary.pdf]

Supplementary Materials

Table S1. Description of the models

The starting point is the Henderson's model in its general form:

$$\mathbf{y} = \mathbf{Xb} + \mathbf{Zu} + \mathbf{e}.$$

The multiple trait (feature) model can be represented as follows:

$$\begin{bmatrix} y_1 \\ y_2 \end{bmatrix} = \begin{bmatrix} X_1 & 0 \\ 0 & X_2 \end{bmatrix} \begin{bmatrix} b_1 \\ b_2 \end{bmatrix} + \begin{bmatrix} Z_1 & 0 \\ 0 & Z_2 \end{bmatrix} \begin{bmatrix} u_1 \\ u_2 \end{bmatrix} + \begin{bmatrix} e_1 \\ e_2 \end{bmatrix}$$

Usually:

$$u = \begin{pmatrix} u_1 \\ u_2 \end{pmatrix} \text{ and } \operatorname{var}(u) = G = \begin{pmatrix} G_{11} & G_{12} \\ G_{21} & G_{22} \end{pmatrix}$$

a 2 by 2 genetic covariance matrix can be defined as:

$$G_0 = \begin{pmatrix} \sigma^2_{g11} & \sigma_{g12} \\ \sigma_{g21} & \sigma^2_{g22} \end{pmatrix}$$

For the hybrid (mixed) model equations, the inverse equation of G is used. It is possible to present a hybrid model with several features as:

$$G^{-1} = \begin{pmatrix} G^{11} & G^{12} \\ G^{21} & G^{22} \end{pmatrix} \text{ where } G^{ij} = g^{ij}A^{-1},$$

The resulting n.2 x n.2 variance-covariance matrix for the total error vector e = (e1, e2)' takes the form as inverted:

$$R^{-1} = \begin{bmatrix} Ir^{11} & Ir^{12} \\ Ir^{21} & Ir^{22} \end{bmatrix}$$

$$\mathbf{R} = \begin{bmatrix} \sigma(e_1, e_1) & \sigma(e_1, e_2) \\ \sigma(e_2, e_1) & \sigma(e_2, e_2) \end{bmatrix} = \begin{bmatrix} Ir_{11} & Ir_{12} \\ Ir_{21} & Ir_{22} \end{bmatrix}$$

A set of equations for a mixed model with multiple features is shown below:

$$\begin{bmatrix} X_1' r^{11} X_1 & X_1' r^{12} X_2 & X_1' r^{11} Z_1 & X_1' r^{12} Z_2 \\ X_2' r^{21} X_1 & X_2' r^{22} X_2 & X_2' r^{21} Z_1 & X_2' r^{22} Z_2 \\ Z_1' r^{11} X_1 & Z_1' r^{12} X_2 & Z_1' r^{11} Z_1 + g^{11} A^{-1} & Z_1' r^{12} Z_2 + g^{12} A^{-1} \\ Z_2' r^{21} X_1 & Z_2' r^{22} X_2 & Z_2' r^{21} Z_1 + g^{21} A^{-1} & Z_2' r^{22} Z_2 + g^{22} A^{-1} \end{bmatrix} \begin{bmatrix} b_1 \\ b_2 \\ u_1 \\ u_2 \end{bmatrix} = \begin{bmatrix} X_1'(r^{11} y_1 + r^{12} y_2) \\ X_2'(r^{21} y_1 + r^{22} y_2) \\ Z_1'(r^{11} y_1 + r^{12} y_2) \\ Z_2'(r^{21} y_1 + r^{22} y_2) \end{bmatrix}$$

The idea is to represent the mixed model as a hybrid model based on single trait (feature) equations. The results presented in the following figures.

Table S2. Structure of the SK-5 diet, which was received by the piglets, who reached a live weight of 20-25 kg (they received the SK-5 diet, when put on rearing).

Parameter	Content
Corn**, %**	45.4
Soy cake**, %**	22.6
Wheat**, %**	19.0
Envelope concentrate 2*, **%**	10.00
Fish meal, %	2.40
Sunflower oil, %	0.40
Salt, %	0.02
Result,%	100.0
**the SK-5 diet included**	
Total Protein, %	21.21
Total fat, %	4.95
Fiber , %	3.00
Lysine, %	1.30
Methionine + cystine, %	0.84
Threonine , %	0.96
Tryptophan , %	0.23
Valin , %	0.92
Choline , mg/kg	909.81
Calcium , %	0.81
Phosphorus, %	0.071

Table S3. Structure of the SK-6 diet, which was received by boards with a live weight from 28-30 kg to 70 kg, (the 1-st period of their fattening).

Parameter	Content
Corn**, %**	37.42
Wheat**, %**	32.50
Soy cake**, %**	19.50
Fish meal**, %**	3.30
Convet 2*, %	1.00
Sunflower oil**, %**	0.40
Salt**, %**	0.26
Sunflower cake, %	4.70
Monocalcium Phosphate, %	0.92
Result, %	100.00
**the SK-6 diet included**	
Total Protein, %	18.31
Total fat, %	4.29
Fiber , %	3.82
Lysine, %	1.15
Methionine + cystine, %	0.61
Threonine , %	0.74
Tryptophan , %	0.20
Valin , %	0.80
Choline , mg/kg	710.30
Calcium , %	0.92

Phosphorus, %	0.87

Table S4. Structure of the SK-7 diet, which was received by boards with a live weight from 70 to 105 kg (the 2nd period of their fattening).

**Parameter**	**Content**
Corn**, %**	23.36
Wheat**, %**	52.00
Soy cake**, %**	12.00
Sunflower cake, %	7.30
Convet 2*, %	1.00
Salt, **%**	0.26
Fish meal**, %**	3.20
Monocalcium Phosphate, %	0.88
Result, %	100.0
**the SK-7 diet included**	
Total Protein, %	17.01
Total Fat, %	3.22
Fiber , %	4.00
Lysine, %	1.03
Methionine + cystine, %	0.60
Threonine , %	0.68
Tryptophan , %	0.19
Valin , %	0.74
Choline , mg/kg	575.70
Calcium , %	0.89
Phosphorus , %	0.87

Figures 1S - 3S.

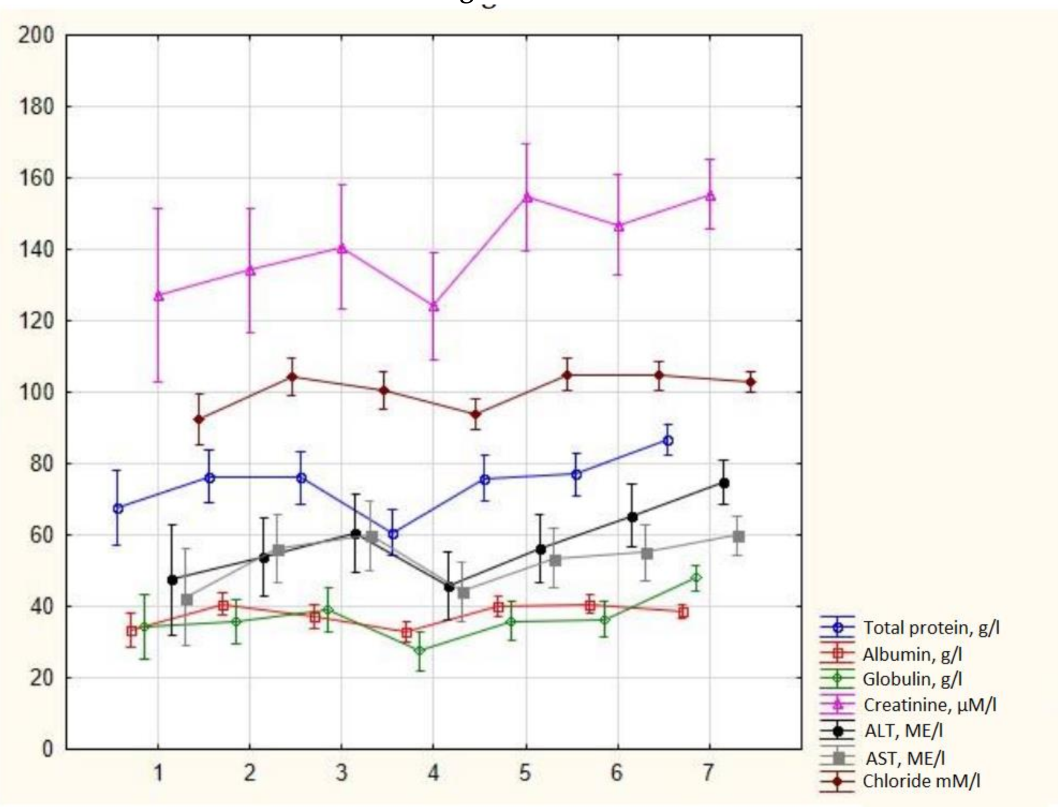

Figure S1. Decomposition of average values of biochemical parameters depending on the influencing factor (FFG).

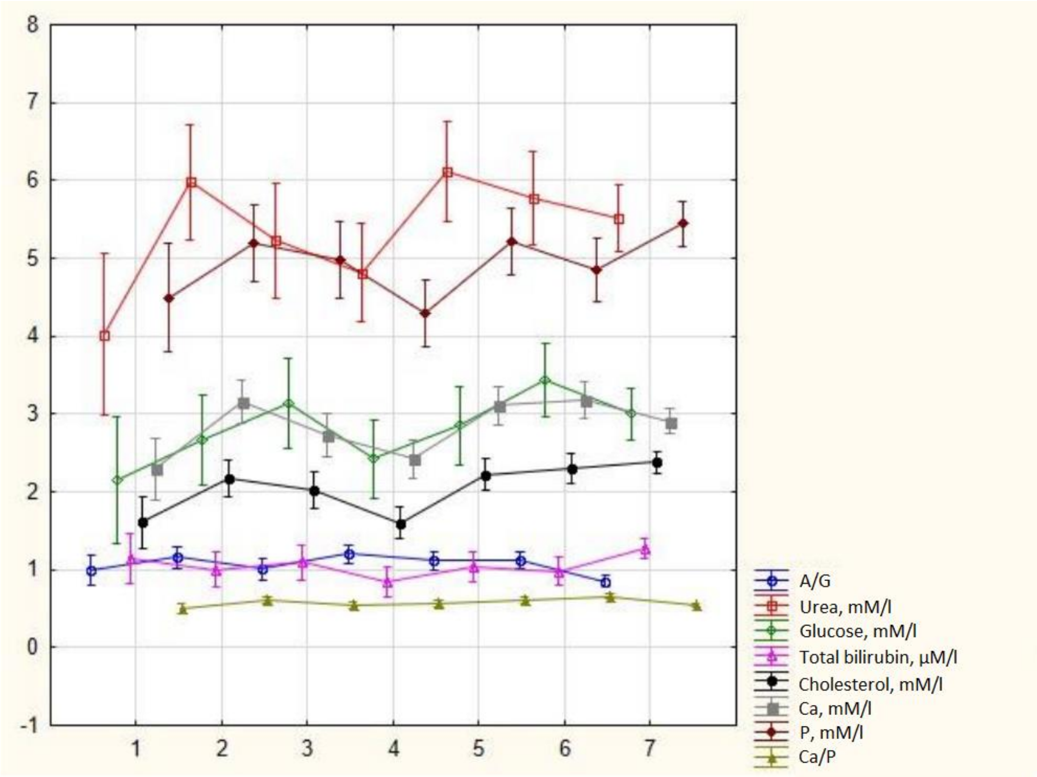

Figure S2. Decomposition of average values of biochemical indicators depending on the influencing factor (FFG).

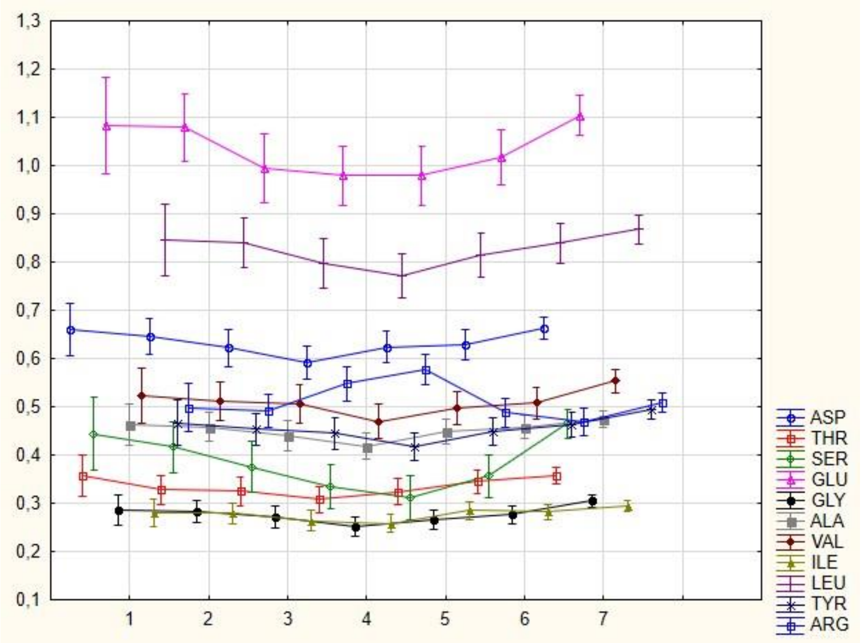

Figure S3. Decomposition of the average values of the amino acid composition of blood proteins depending on the influencing factor (FFG).
